# Nanobiotechnology augmented cancer stem cell guided management of cancer: liquid-biopsy, imaging, and treatment

**DOI:** 10.1186/s12951-024-02432-5

**Published:** 2024-04-12

**Authors:** Si Sun, Qiang Yang, Dawei Jiang, Yuan Zhang

**Affiliations:** 1 Department of Obstetrics and Gynecology, Union Hospital, Tongji Medical College, Huazhong University of Science and Technology, Wuhan, 430022 China; 2 Department of Nuclear Medicine, Union Hospital, Tongji Medical College, Huazhong University of Science and Technology, Wuhan, 430022 China; 3 Hubei Key Laboratory of Molecular Imaging, Wuhan, 430022 China; 4 Key Laboratory of Biological Targeted Therapy, the Ministry of Education, Wuhan, 430022 China

**Keywords:** Cancer stem cell, Nanobiotechnology, Circulating tumor DNA, Molecular imaging, Liquid biopsy, Nuclear medicine

## Abstract

Cancer stem cells (CSCs) represent both a key driving force and therapeutic target of tumoral carcinogenesis, tumor evolution, progression, and recurrence. CSC-guided tumor diagnosis, treatment, and surveillance are strategically significant in improving cancer patients’ overall survival. Due to the heterogeneity and plasticity of CSCs, high sensitivity, specificity, and outstanding targeting are demanded for CSC detection and targeting. Nanobiotechnologies, including biosensors, nano-probes, contrast enhancers, and drug delivery systems, share identical features required. Implementing these techniques may facilitate the overall performance of CSC detection and targeting. In this review, we focus on some of the most recent advances in how nanobiotechnologies leverage the characteristics of CSC to optimize cancer diagnosis and treatment in liquid biopsy, clinical imaging, and CSC-guided nano-treatment. Specifically, how nanobiotechnologies leverage the attributes of CSC to maximize the detection of circulating tumor DNA, circulating tumor cells, and exosomes, to improve positron emission computed tomography and magnetic resonance imaging, and to enhance the therapeutic effects of cytotoxic therapy, photodynamic therapy, immunotherapy therapy, and radioimmunotherapy are reviewed.

## Background

Cancer stem cells (CSCs) are a small group of cancer cells with stem-like properties. They have the capabilities of self-renewal, differentiation, and hence tumorigenicity. Compared to common cancer cells, CSCs are often in a quiescent state, express an elevated level of drug efflux pumps, have a higher capacity for DNA repair as well as anti-apoptosis, can cross-talk with tumor microenvironment to build up shields for immune escape and in turn remodeled by tumor microenvironment to undergo epithelial-mesenchymal transition. Due to these characteristics, CSCs are less vulnerable to chemotherapy and radiotherapy and, therefore, become one of the major causes of cancer recurrence and metastasis. Targeting CSC is a rational strategy to detect early-stage cancers, reverse chemotherapy and radiotherapy resistance, and prevent metastasis.

However, targeting CSC is challenging not only due to their innate characteristics, including rarity and resistance to chemotherapy and radiotherapy but also due to their heterogeneity and plasticity [1]. CSCs are a group of cells with high intertumoral and intratumoral heterogeneities. CSCs from different types of tumors may arise from tissue-specific or pluripotent stem cells. Within a given tumor, CSCs can exhibit heterogeneity in terms of their phenotypic characteristics, such as their expression of cell surface markers, their sensitivity to chemotherapy or radiation, and ability to form metastases. This heterogeneity can arise from genetic mutations or epigenetic modifications during tumor evolution. CSCs can also exhibit plasticity, meaning they can switch between stem cell and non-stem cell states or phenotypes in response to changes in their microenvironment [2].

**Fig. 1:**
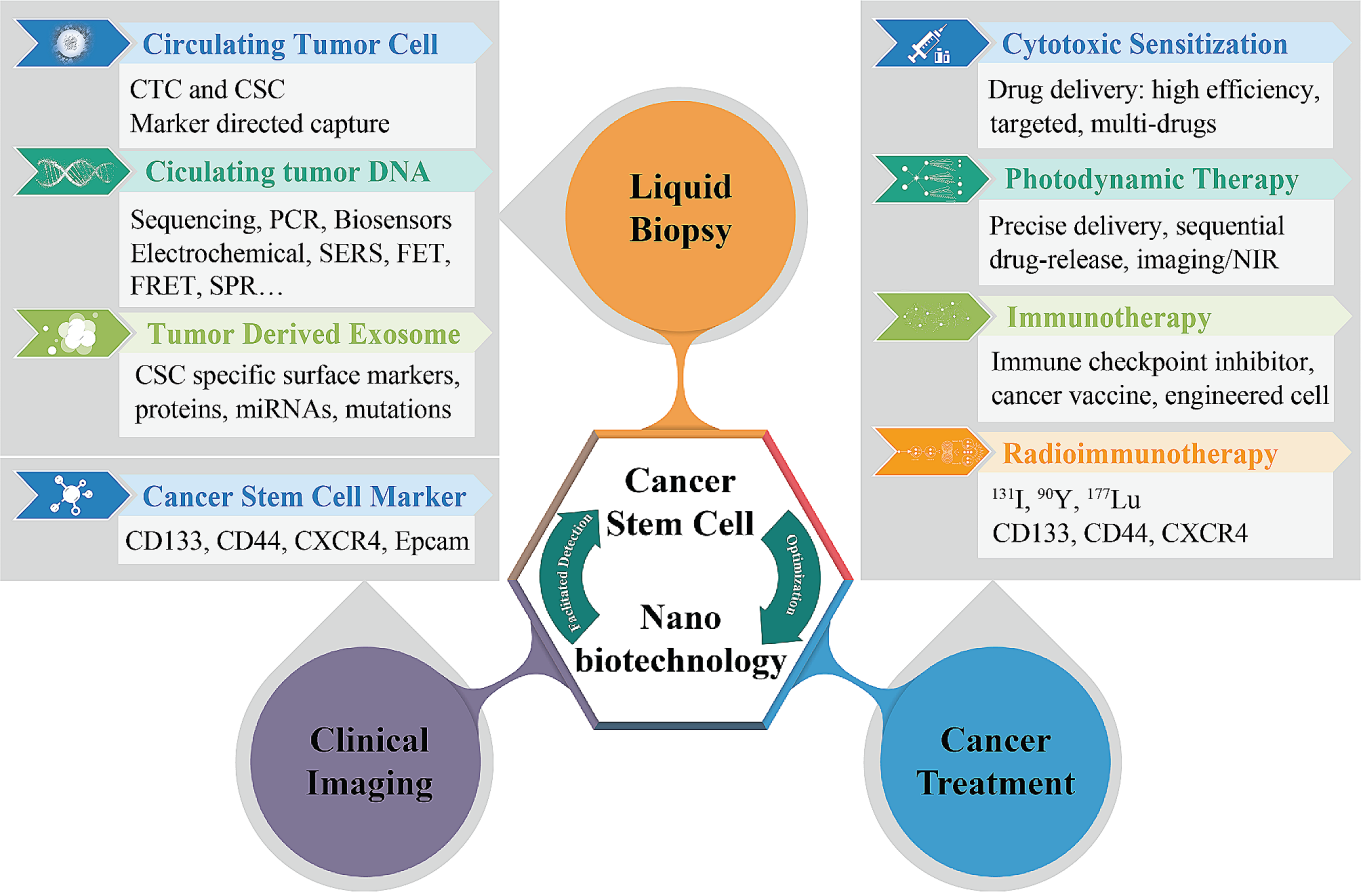
Leveraging characteristics of cancer stem cells for liquid biopsy, clinical imaging, and cancer treatment

Compared to conventional methods aiming at CSC-targeting, nanobiotechnology offers several advantages in cancer diagnosis, imaging, and treatment. For cancer diagnosis, nanomaterials can be engineered to bind specific surface cancer stem cell markers and trigger signal amplification. For imaging, nanomaterials can be used as contrast agents for more precise tumor detection. For cancer treatment, nanobiotechnology-engineered drug delivery systems have considerable advantages in targeted drug delivery, enhanced drug permeation and retention, reduced toxicity, multi-functionality, and personalization. Previous reviews have introduced how nanobiotechnologies exploited CSC characteristics, including cell-surface markers, metabolism, and microenvironment for biological imaging and chemotherapeutic enhancement [3, 4]. In this review, we focus on some of the most recent advances in how nanobiotechnologies leverage the characteristics of CSC to optimize cancer diagnosis and treatment, specifically in liquid biopsy, positron emission computed tomography (PET) and magnetic resonance imaging (MRI), cytotoxic therapy, photodynamic therapy, immunotherapy therapy and radioimmunotherapy (Fig. 1).

### Nanobiotechnology-optimized CSC-directed liquid biopsy

**Fig. 2:**
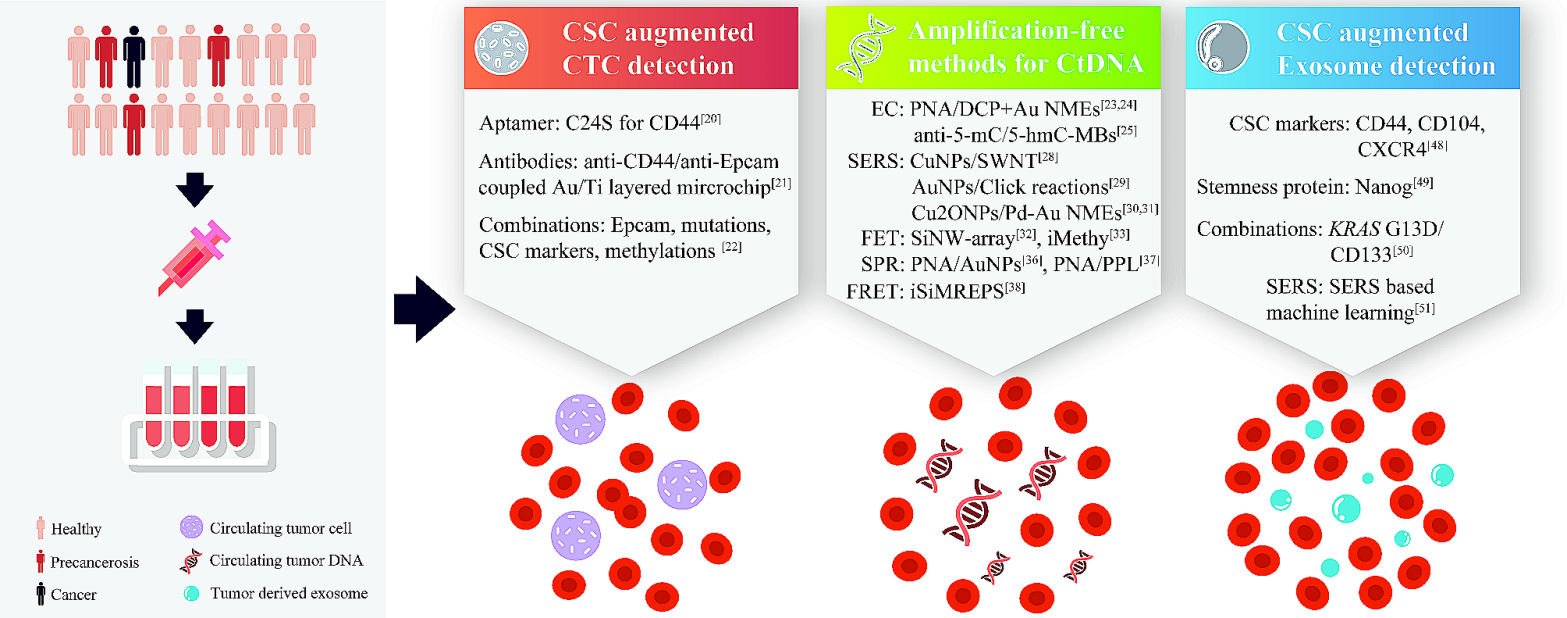
Nanobiotechnology-modified circulating tumor cell, tumor DNA, and tumor-derived exosome detection

Liquid biopsy is a diagnostic test that involves the analysis of body fluids. In tumor diagnosis and monitoring, the test detects the presence of circulating tumor cells (CTCs), cell-free circulating tumor DNA (ctDNA), circulating tumor-derived exosome, and other biomarkers in the blood, which can provide valuable information about tumor volume and disease status. Compared to tissue biopsy, liquid biopsy is non-invasive, practically repeatable, and can provide more systematic information disregarding intra-tumor and inter-tumor heterogeneity. Real-time information from liquid biopsy may offer promising opportunities for cancer diagnosis and treatment monitor. However, several limitations hindered the clinical application of liquid biopsy: (1) super-low concentration of CTCs and ctDNA in early-stage disease and certain types of cancer; (2) demand for more specific markers; (3) high-cost and turnover time for sequencing. In this part, we mainly review the current statuses of CTC, ctDNA, and circulating tumor-derived exosome detection and how characteristics of CSC are exploited for nanobiotechnology-modified optimizations (Fig. 2).

#### Circulating tumor cell

CTCs are cancer cells detached from primary or metastatic tumor lesions that enter the blood or lymphatic system to travel along the circulation system and potentially form new tumors. The clinical significance of CTCs lies in their potential use as a biomarker for cancer diagnosis, prognosis, and treatment response monitoring. Studies have shown that the number of CTCs in a patient’s blood is associated with disease progression and poor prognosis in various types of cancer, including breast, lung, colorectal, and prostate cancer [5, 6]. Additionally, CTCs can provide information about the genetic and molecular characteristics of the tumor, which can help guide treatment decisions. Therefore, CTC analysis is one of the most important paradigms of liquid biopsy.

CTCs are detected in various types of cancer, including breast cancer, NSCLC, colorectal cancer, lung cancer, and ovarian cancer. The common criteria to distinguish CTC from normal cells were cell diameter, shape, and molecular markers. (> 9 μm) and specific surface markers (CD45 negative to exclude leukocyte contamination). A series of platforms have been launched for CTC detection in clinical and research settings: CellSearch, the first and the only Food and Drug Administration (FDA) approved CTC test, detect CTCs in patients with breast, colorectal, and prostate cancer (EpCAM^+^, cytokeratin 8^+^, 18^+^ and/or 19^+^ and CD45^−^); Epic Sciences capture CTCs and classify them based on molecular markers (EpCAM, cytokeratins, HER2, androgen receptors, etc.) and morphological features including size and shape. However, CTC detection is still a relatively new and developing technology. Current CTC tests may have some potential drawbacks, including limited sensitivity, lack of specificity, high cost, and limited availability.

CTCs and CSCs are two groups of cancer cells with crucial clinical significance sharing overlapping properties. They may both undergo epithelial-mesenchymal transition and enhanced trans-endothelial migration. Although a higher number of CTCs detected in the periphery blood was associated with a higher risk of metastasis, only a limited fraction of CTCs can successfully manage to initiate metastatic tumor lesions. Accumulated evidence strongly suggested the existence of CSCs among CTCs: CTC cell lines derived from chemotherapy-naive stage IV colorectal cancer patients maintained with non-serum suspension culture condition had the strong tumor-initiating capacity and were able to differentiate into main intestinal lineages both in vitro and in vivo [7]; compared to colorectal cancer xenograft-derived organoid, CTC-derived organoid of the same xenograft presented increased stem cell marker expression [8]; genetic linage tracing of stemness activation of murine glioblastoma (GBM) CTCs with native microenvironment presented nearly 100% of SOX2 activation, while less than 5% of primary tumor cells were SOX2 activated. Additionally, almost all murine GBM CTCs expressed Olig2, and 40% expressed CD133 [9]; CSC markers are detected on a fraction of CTCs in a variety of types of cancer, including breast cancer (CD44), colorectal cancer (LGR5), non-small cell lung cancer (NSCLC) and ovarian cancer (BMI1, CD133, ALDH1) [10–12].

To achieve circulating CTC detection, there were several challenges to overcome: the rarity of CTCs from a draw of blood, the innate heterogeneity of CTCs, and the lack of standardized CTC detection methods. Circulating CSCs are evolving as potential biomarkers in combination with CTCs for liquid biopsy. The combination of the CTC and CSC markers presented significant prognosis-predicting value in colorectal cancer [13]. Compared to CSC marker negative CTC, detection of CSC marker positive CTC was associated with metastases, increased risk of relapse and death in late-stage breast cancer [14, 15], NSCLC [16], prostate cancer [17], pancreatic cancer [18] and ovarian cancer [19]. The addition of CSC marker-directed cell sorting would increase the heterogeneity of CTC detected by unitary EpCAM-directed cell sorting, enhancing the clinical significance of CTC detected. Therefore, taking advantage of circulating CSC identification might boost the development of CTC detection in clinical settings. Similar challenges remain for circulating CSC detection due to their rarity, heterogeneity, and plasticity. The application of nanobiotechnology is apt to increase circulating CSC detection by enhancement of CSC capture using nanomaterials with appropriate charge, magnetism, or binding affinity to CSC-specific antigens.

A few nanobiotechnology-augmented CTC detection strategies based on CSC marker detection achieved outstanding performance in human sample testing. Gao et al. screened out and modified a CD44 targeting aptamer C24S for preparation of C24S conjugated magnetic nanoparticles, which greatly enhanced CD44 targeting specificity and enabled easy isolation of CD44^+^ circulating cells using magnet [20]. Kwizera et al. designed a gold-titanium coupling layer-coated electro-microfluidic chip with a linkage of heterogeneous CTC capture antibodies, including anti-CD44 and anti-Epcam. Compared to single marker-directed CTC capturing, a combined capture strategy of multiple subtypes of CTCs did not hamper the capture purity [21]. Their work provided a conceivable solution to improve the coverage of CTC and decrease the false negative rate due to CTC heterogeneity. Stergiopoulou et al. reported a long-term follow-up of 13 breast cancer patients who received comprehensive CTC monitoring, including measurements of EpCAM, phenotypic analysis, CSC marker analysis, *PIK3CA* and *ESR1* mutations, and *ESR1* methylation. According to their study, all patients with at least one positive marker relapsed, and all patients with negative markers stayed progression-free during follow-up [22]. Although the cohort examined by Stergiopoulou et al. was small and the CTC detection strategy applied was not integrated, a comprehensive CTC detection strategy consisting of multiple measurements might help to improve the detection rate of circulating CSC compromised by CSC plasticity.

#### Circulating tumor DNA

Cell-free DNAs were fragments from apoptotic cells that shed into body fluids. CtDNAs were cell-free DNAs from apoptotic or necrotic cancer cells. CtDNA analysis can detect tumor-associated features, including tumor-specific mutations, structural variants, copy number alterations, epigenetic features, microsatellite instability status, gene expression patterns, and tumor heterogeneity. CtDNA has several advantages as one of the paradigms of liquid biopsy: the amount of ctDNA was reported to be associated with tumor burden, which makes ctDNA testing a promising way to detect minimally residual disease; the genetic alteration of ctDNA represents tumor mutation burden and therefore could be used as a potential predictive biomarker for treatment response to immune checkpoint inhibitors; the sequential monitoring of specific somatic genetic mutations from the primary tumor through ctDNA sequencing may provide dynamic evidence for predictive treatment response to specific targeted therapies. In recent years, the FDA cleared several ctDNA-based tests, including *EGFR*, *KRAS*, *BRAF*, and *EZH2* mutation analyses for customized targeted treatment planning. Although not yet cleared by the FDA, the device for Natera’s ctDNA tests was granted breakthrough device designation. These breakthroughs powerfully drove the development of novel methods and technologies to improve the sensitivity, specificity, and accuracy of ctDNA analysis.

DNA sequencing and amplification-based approaches are the mainstream and currently the gold-standard technology of un-informed and informed ctDNA detection. However, long turnover, the high cost of DNA sequencing, and the requirement of specialized pretreatment of samples for amplification tests limited clinical ctDNA application. For informed ctDNA testing, low-cost, point-of-care, and pretreatment-free technology is urgently needed. Nanobiotechnology has been implemented in several aspects of ctDNA detection to achieve amplification-free, super-sensitive, low-cost, and real-time ctDNA detection. The most promising strategies include fluidics-based electrochemical sensors, fluidics-based surface-enhanced Raman scattering (SERS), surface plasmon resonance (SPR), field-effect transistors (FETs), and Förster resonance energy transfer (FRET) based nano-sensors.

Electrochemical sensors are one of the most frequently studied solutions to ctDNA detection due to their low cost, simplicity, portability, and robustness. Constant endeavors boosted continuous optimizations of the performance of electrochemical sensors in ctDNA detection from multiple monogenic single point-mutation detection by peptide nucleic acids capture combined with Si3N4/Au/Si nanostructured microelectrodes [23] to multiple hotspot mutation detection via combinational probes [24], from double-strand DNA detection [25] to methylated DNA detection [26]. In this field, the low detection limit is pushed to 10 aM and even lower [27].

SERS is another powerful way to detect ctDNA at a single molecular level. Qi et al. developed a SERS probe using single-walled carbon nanotubes, which detected *KRAS* G12DM as low as 0.3 fM in an aliquot of 0.5 µl sample [28]. To fulfill multiplex detection, Yi et al. synthesized several SERS probes with remarkably different single-band Raman scattering signals, allowing a 10-plex biomarkers detection [29]. Cao et al. developed ultrasensitive SERS probes Cu_2_O@DTNB@hp3-1/Cu_2_O@DTNB@hp4-1 (PIK3CA E542K), Cu_2_O@4-ATP@hp3-2/Cu_2_O@4-ATP@ hp4-2 (TP53), Pd-AuNRs@DTNB@HP1-1 (BRAF V600E) and Pd-AuNRs@4-MBA@HP1-2 (KRAS G12V) with translational potential in diagnosis and follow-up of NSCLC patients. They employed Cu_2_O octahedra and Pd-Au nanorods for modification of SERS reporters to enhance field coupling, on-chip capillary pump for exemption of external pumps and rapid mixing, catalytic hairpin assembly for non-enzymatic signal amplification [30, 31].

FETs are relatively novel biosensors for ctDNA detection. Du et al. developed a silicon nanowire array field-effect transistor biosensor for *PIK3CA* E542K detection. The sensor achieved an ultralow detection limit of 10 aM and a good linearity under the ctDNA concentration range from 0.1 fM to 100 pM [32]. Pei et al. constructed a wearable self-healing patch consisting of a 3D printed eutectic gallium-indium circuit that can endure 100% strain and a FET sensor (iMethy) functionalized with 5-methylcytosine monoclonal antibody for specific methylated ctDNA capture and detection. The FET sensor can detect ctDNA as low as 0.1 fM in vitro, distinguishing tumor-bearing mice from healthy controls [33]. These findings suggested the translational potential of FET ctDNA biosensors as dynamic point-of-care ctDNA detection devices.

SPR is another frequently reported method of ctDNA detection based on real-time and quantitative information. Pioneering studies laid the foundation of gold nanoparticle-based plasmonic bio-sensors for detecting DNA sequences [34, 35]. Recent works aim at both DNA sequence variation and DNA methylation detection. Anh et al. developed a SERS-based nano-plasmonic biosensor for E542K and E545K mutations of *PIK3CA* and immunogold colloids for two methyl-cytosines in the promoter region with detection concentration of 200 fM [36]. Noemi et al. exploited a functional poly-L-lysine-based surface layer and plasmonic biosensor to detect the binding of MBD2 to methylated DNA. Their detection method had a high sensitivity (0.1 pg/mL, approximately 1 fM if the length of ctDNA is considered), specificity (99.6%), and accuracy (99.4%) for detecting methylated DNA in human plasma samples from healthy donors and colorectal cancer patients [37].

Rapid fingerprint single molecular detection of ctDNA through FRET-based nano-sensors was also feasible. Kunal et al. designed a DNA sensor consisting of fluorescent-tagged capture and query probes. The capture of mutated target ctDNA would shorten the physical distance between the capture and the query probe, thus triggering FRET. The FRET ctDNA probes could detect two biomarkers in 10 s with a detection limit of 3 fM and a mutant fraction as low as 0.0001%. However, single-molecule fluorescence microscopy, which is not commonly available in usual clinical settings, is required for FRET signal identification [38].

The underlying rationale for the identification of ctDNA of CSC from total ctDNA can be addressed in the following aspects: CSC is considered as one of the drivers of tumor initiation, progression, metastasis, and resistance to therapy hence positive ctDNA of CSC may provide a more accurate reflection of tumor burden, metastatic potential and prediction of treatment response; the detection of ctDNA from CSCs may allow for earlier detection of recurrence, timely treatment, and improved patients’ outcomes; CSCs’ competence of handling intrinsic and extrinsic hazards guarantees the longevity of the population to accumulate and preserve incremental genetic features; considered as one of the main driving forces of tumor evolution that give rise to phenotypic tumor heterogeneity, a pool of CSC was hypothesized as a representation of overall tumor heterogeneity that enables the capture of tumor heterogeneity [39–41].

Biosensors targeting CSC-derived ctDNA were studied for early diagnosis and disease surveillance. Ashok et al. designed nano-engineered plasmonic meta-sensors for real-time Raman scattering mapping of DNAs from glioblastoma cells, cell-line derived glioblastoma stem cells, tumor samples, and serum. The DNA data were then incorporated with machine learning for glioblastoma diagnosis. The addition of glioblastoma stem cell data to machine learning training significantly improved the sensitivity (83.3–93.3%) and specificity (75–100%) of the algorithm [42]. The same team also constructed a quantum superstructure to enhance SERS and mapped the SERS signal spectrum of DNA from breast, lung, and colorectal cancer cells, their cancer stem cell counterparts as well as corresponding tumor samples. With the Raman signals of cancer cells and cancer stem cell DNA as training data, ctDNA detection was achieved in raw plasma samples with 97% sensitivity and 83% specificity. With the addition of CSC DNA data, the algorithm distinguished lung cancer from mixed cancers with a sensitivity of 83.33% and 96.15%. With tumor DNA profile supplemented with the training, the algorithm further distinguished lung cancer from breast cancer and colorectal cancer, providing proof-of-concept evidence that tumor origin identification could be achieved through SERS-based liquid biopsy. CSC-derived ctDNA was an indispensable measurement [41].

#### Circulating tumor-derived exosome

Exosomes are nano-sized (30 to 50 nm) vesicles containing various types of biomolecules, including proteins, lipids, and nucleic acids released by cells for cell-cell communication. Exosomes participate in various physiological and pathological processes, including immune response, inflammation, cancer, and neurological disorders. Compared to CTC and ctDNA, tumor-derived exosomes had several advantages as a liquid biopsy detection alternative. Exosomes are much more abundant and stable than CTC and ctDNA in the blood, which potentiate sample acquisition. Unlike CTC and ctDNA, which were shed from bulk tumors at later stages or apoptotic tumor cells, exosomes were secreted by tumor cells throughout the tumor initiation and progression phase. However, there were still some limitations regarding exosome testing in liquid biopsy. The primary two were the lack of standardized exosome isolation, characterization, and quantification methods and the difficulty distinguishing tumor cell-derived exosomes from normal cell-derived exosomes. The most commonly used surface markers to identify exosomes were CD9, CD63, CD81, CD82, CD37, CD53, CD151, ALIX, and HSP70 [43]. However, exosomes were quite heterogeneous groups, and it was suggested that exosomes would acquire a fraction of surface markers from their cell of origin. For example, exosomes derived from dendritic cells may express CD86 and CD83, and exosomes from B cells may express CD19 and CD20 [44].

The biological characteristics, function, and destination of exosomes are different based on the cell of origin, cargos contained, and surface markers [45]. Compared to cancer cell-derived exosomes, CSC-derived exosomes had a unique role in the CSC plasticity remodeling [46]. They were more intimately involved in the promotion of cancer cellular epithelial-mesenchymal transition Field [47], therapeutic resistance Field [48], angiogenesis, and immune interaction [47]. Therefore, detecting CSC-derived exosomes might reflect cancer relapse, dynamic progression, therapeutic resistance, etc. Unlike non-cancerous tissue-derived exosomes and cancer cell-derived exosomes, CSC-derived exosomes contained cancer stem cell-specific surface markers, stemness-specific proteins, self-renewal regulatory microRNAs, and cancer stem cell-specific mutation signatures. Major pancreatic stem cell markers, including CD44v6, CD104, and CXCR4, were enriched in corresponding pancreatic stem cell-derived exosomes [48]. Stemness-specific protein Nanog was detected in exosomes from high-grade serous ovarian cancer patients [49]. Oncogenic *KRAS* and *KRAS* G13D carrying CD133^+^ microvesicles derived from colorectal cancer were proven to promote chemotherapy resistance [50]. These specific features of cancer stem cell-derived exosomes may serve as biomarkers for cancer-specific exosome isolation and improve sensitivity and specificity for cancer diagnosis and monitoring when combined with the current liquid biopsy strategy.

Rupa et al. exploited a machine learning algorithm in combination with SERS data to detect CSC-derived exosomes from blood samples. They collected exosomes from fibroblasts, MDAMB231 (breast cancer), H69AR(lung cancer), COLO205 (colorectal cancer), and their CSC counterparts. The samples were examined by SERS, the signal amplified by their nanosensor, and the data was used for artificial neural network training. After adjustment based on non-cancerous signals, SERS data of CSC-derived exosomes, containing crucial signals for cancer diagnosis, turned out to be different from cancer cell-derived ones. The machine learning algorithm was able to distinguish cancer samples from the non-cancer samples with 100% sensitivity and 100% specificity and identify tumor origin (breast cancer, sensitivity 73%, specificity 88%; colorectal cancer, sensitivity 42%, specificity 100%; lung cancer, sensitivity 100%, specificity 70%) [51].

### Nanobiotechnology-optimized CSC-directed imaging

CSC-directed imaging is an indispensable technique that helps to identify the rare population of CSCs responsible for tumor initiation, progression, metastasis, therapeutic resistance, and recurrence. The prospect of clinical application of CSC imaging is extensive, not limited to understanding CSC evolution, tumor metastasis evaluation, treatment response monitoring, prognostic outcome prediction, and novel anti-tumor strategy development. According to the general characteristics of CSCs, two major strategies for CSC imaging were commonly exploited: targeting CSC markers, CSC-featured pathways, or metabolic activities. Theoretically, CSC imaging would allow for a thorough evaluation of systemic dynamics and heterogeneities of CSCs in different sites and microenvironments, which could exempt the patients from successive painful histologic biopsies. CSC imaging is still a developing field that faces numerous challenges and limitations.

Nanoscale materials and technologies engineered by nanobiotechnology facilitated biological and clinical purposes in many ways, including bioimaging. Target-specific nanocarriers precisely delivered fluorescent, magnetic, and radioactive contrast to the tissue of interest [52]. Modified nanocarriers and nanosensors could amplify rare and otherwise undetectable signals. Nanobiotechnology can thus enable more accurate and non-invasive monitoring of rare populations like CSCs. Since CSCs were generally identified through acknowledged CSC markers but not CSC-associated pathways, here, we mainly focused on reviewing the most acknowledged CSC marker-directed imaging and corresponding nanobiotechnology-driven strategies.

#### CD133

CD133 is a penta-span membrane glycoprotein and one of the most well-acknowledged CSC markers in various types of cancer. Preclinical and proof-of-concept studies on PET imaging probes for targeting CD133 tumor cells have been performed in glioma, prostate, lung, liver, colorectal, and many other tumor models. Namely, Gaedicke et al. reported noninvasive PET imaging of orthotopic CD133^+^ xenografts and patient-derived CD133^+^ CSCs by ^64^Cu-NOTA-AC133 mAb [53]. Glumac et al. conjugated PET probe ^89^Zr-HA10 IgG for CD133^+^ tumor imaging and validated its specificity using CD133^+^ and CD133^−^ aggressive variant prostate cancer tumor-bearing mice. Hu et al. screened out a CD133-directed ^64^Cu PET tracer ^64^Cu CM-2 and tested its imaging performance and specificity in human hepatoma, melanoma, glioblastoma, breast cancer (Huh-7, Bowes, U87MG, MDA-MB231) and murine melanoma B16F10 allografts [54]. While a PET scan was unavailable, CD133^+^ tumor imaging could be achieved through specific CD133 contrast enhancement agents through MRI imaging. Chen et al. designed a CD133 antibody conjugated ultrasmall superparamagnetic iron oxide (USPIO) for a specific signal reduction in both FSE T2-weighted and merged scenarios. In addition to specific imaging of CD133^+^ HT29 subcutaneous xenografts, USPIO-CD133 Ab could also delineate drug-induced murine brain tumors [55].

A few studies tested the feasibility of CD133-directed testing as indicators for early tumor detection. Based on the findings that CD133 was explicitly expressed in small cell lung cancer (SCLC) but not in other types of lung cancer, such as adenocarcinoma and squamous cell carcinoma or adjacent healthy tissues, Kunihiro et al. proposed a strategy for early detection of SCLC that involved CD133 based PET imaging and autoantibody detection. They synthesized a CD133 targeting probe ^89^Zr-DFO-αCD133, which reached tumoral uptake of more than 40% of the injected dose in a xenograft mouse model of SCLC. Additionally, they found that αCD133 autoantibody could be found within one year pre-SCLC-diagnosis in 20/31 SCLC patients but not 55 controls consisting of colon, pancreas, and non-SCLC patients from the cardiovascular health study and the prostate, lung, colorectal, and ovarian screening trial [56]. Although the implementation of PET scan as a screening test was not feasible, constrained to cost, equipment availability, and complexity of the procedure, the findings of Kunihiro et al. allow us to posit a reasonable three-step strategy for early tumor detection: regular blood test as the initial screening, PET-scan for added evidence in high-risk patients and then surgical biopsy for diagnosis and treatment.

In addition to early tumor detection, treatment monitoring by CD133-directed imaging was also evaluated preclinically. Jung et al. found that celecoxib might have a CD133 modulation effect, so they synthesized a PET probe ^89^Zr-DFO-AC133.1, which targeted the glycosylated epitope of CD133, validated its CD133 targeted imaging specificity in human colon adenocarcinoma HT29 xenografts and tested six-day short-term CD133 level monitoring post-celecoxib-treatment [57]. Clinical evaluation of CD133-directed imaging had not been carried out in humans, possibly due to technique limitations and rationale deficiency. On one hand, there is currently no FDA-approved CD133 targeted therapy, which does not prioritize the need for CD133-directed imaging. On the other hand, the specificity, stability, and safety of current pre-clinical CD133-targeted PET tracers need further improvement. Several CD133 agents were under investigation in clinical trials, and hopefully, this result might boost the development and clinical translation of CD133-directed imaging.

#### CD44

CD44 is one of the earliest and most commonly acknowledged CSC markers intimately involved in tumor invasion and metastasis. Due to alternative splicing, the CD44 coding gene generated several different exon-containing variants, including CD44v3, CD44v4, CD44v6, and CD44v8-10, in addition to a standard form of CD44. According to some researchers, CD44 variants were also considered promising cancer biomarkers. The functional involvement of CD44 and its variants in tumor progression made them both CSC markers and therapeutic targets. Hence, CD44 targeted imaging could be used to select patients for CD44 targeted therapy, determine the dose for radioimmunotherapy, or monitor treatment response.

Since 2007, sustained efforts have been made to strive for the clinical translation of CD44-targeted PET imaging from lab studies. Several humanized anti-CD44 and anti-CD44 variant monoclonal antibodies including bivatuzumab and RG7356 or RO5429083 were selected for non-invasive imaging of CD44 + tumors and tested in phase 1 clinical trials of breast cancer (NCT02254005), head and neck neoplasms (NCT02254018), and metastatic or locally advanced solid tumors (NCT01358903) [58]. The pioneering clinical investigation of nuclide-labeled bivatuzumab-directed imaging of CD44 + tumors seemed halted due to the severe side effects during bivatuzumab treatment. Although the phase I dose escalation study of RG7356 was not interrupted by safety concerns, both specific and non-specific uptake of RG7356 in normal tissues limited the use of RG7356 in a fashion of antibody-drug conjugate [59]. Considering that smaller molecular weight might allow for higher tumor penetration capacity, Philipp et al. screened out a single-chain fragment variable with high affinity to CD44. They tested the ability of CD44-directed PET imaging in the form of bivalent antibodies labeled with ^64^Cu and ^89^Zr [60]. The probe ^89^Zr-DFO-scFv-Fc-CD44 demonstrated outstanding tumor uptake, which warrants the possibility of further clinical evaluation. However, up to now, relevant in-human evidence was still inadequate.

In addition to general CD44-directed imaging, both direct and indirect CD44 variant-directed imaging were also investigated. Haylock et al. constructed a bivalent CD44v6 targeting Fab antibody fragment linked through a self-dimerizing helix-turn helix motif named AbD19384 as the probe base, which was further labeled with ^125^I or ^124^I. ^124^I-AbD19384 showed superior tumor imaging ability than ^18^F-Fluoro-2-deoxy-D-glucose (^18^F-FDG) in CD44 positive breast cancer model MDA-MB-231 bearing mice. The authors’ primary aim was to test the strategy using bivalent Fabs to achieve in vivo imaging, so they did not focus on the overall performance of the CD44v6 imaging [61]. Since a splice variant of CD44 [62] was reported to interact with and stabilize xCT, which was responsible for uptake of a radiotracer (S)-4- (3- ^18^F-Fluoropropyl)-L-Glutamic Acid (^18^F-FSPG), the correlation between tumor uptake of ^18^F-FSPG and system xC/CD44 were examined in a few mini-cohorts of patients with NSCLC, breast cancer, hepatocellular carcinoma and prostate cancer [63–65]. According to these in-human data, tumor uptake of ^18^F-FSPG might be representative of redox status but not necessarily the status of CD44. Therefore, indirect imaging might help understand certain CD44-relevant mechanisms but is not readily available for clinical translation.

#### CXCR4

As a chemokine receptor, CXCR4 is intimately involved in cell survival, proliferation, and migration. It has been found to be associated with patients’ survival in various types of cancer, including leukemia, multiple myeloma, and breast carcinoma. CXCR4 was also engaged in support of CSC properties such as self-renewal, differentiation, and treatment resistance for certain types of cancer through endogenous maintenance and CSC-CSC niche crosstalk mediation. Therefore, CXCR4 is considered a biomarker for radioresistant CSCs and a potential therapeutic target.

**Fig. 3:**
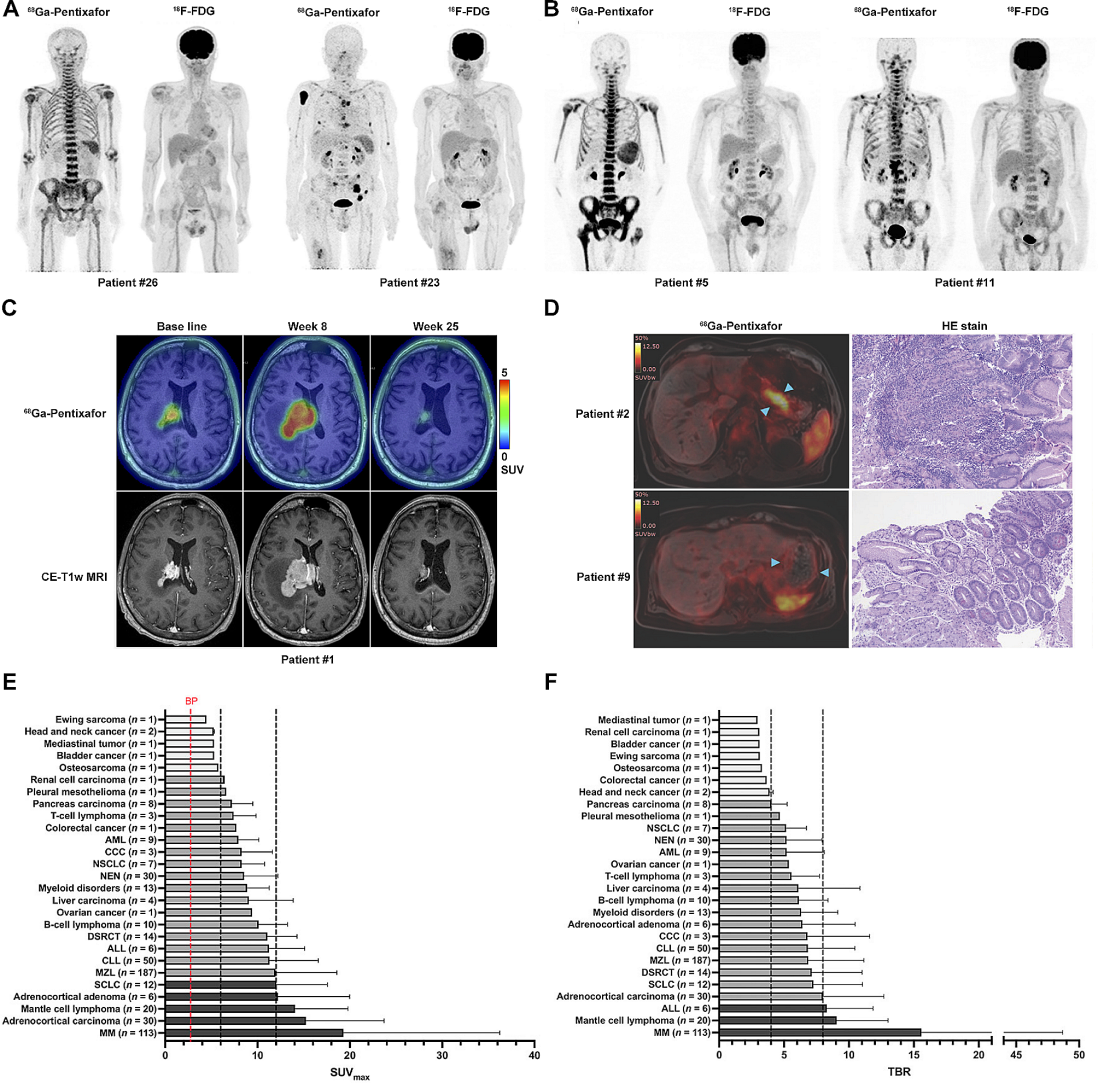
^68^Ga-Pentixafor-PET imaging of hematologic and solid cancer patients. Enhanced uptake of ^68^Ga-Pentixafor in bone marrow tissue in MM patient #26 and bone marrow lesions in MM patient #23 [79]. B Enhanced uptake of ^68^Ga-Pentixafor in bone marrow tissue in recurrent WM patient #5 and multiple lymph nodes of WM patient #11 [81]. C ^68^Ga-Pentixafor PET imaging and corresponding CE-T1w MRI imaging of recurrent CNSL patient #1 at time points of pre-treatment, completed chemotherapy (week 8), and completed radiotherapy (week 25) [85]. D ^68^Ga-Pentixafor PET/MRI imaging and HE staining of post-treatment MALT patients #2 and #9 [90]. E and F The average SUVmax and TBR of ^68^Ga-Pentixafor in solid cancer patients [87]. MM, multiple myeloma; WM, waldenström macroglobulinemia; CNSL, central nervous system lymphoma; NSCLC, non–small cell lung carcinoma; NEN, neuroendocrine neoplasm; AML, acute myeloid leukemia; CCC, cholangiocarcinoma; CLL, chronic lymphocytic leukemia; MZL, marginal zone lymphoma; DSRCT, desmoplastic small round cell tumor; SCLC, small cell lung carcinoma; ALL, acute lymphoblastoid leukemia. All panels were reproduced with permission

Compared to other CSC markers, CXCR4 targeted imaging was one of the most extensively studied, and very few went on to clinical trials. A wide range of CXCR4 tracers has been developed and tested preclinically: ^64^Cu-AMD3100 [66], ^18^F-T140 [67], ^64^Cu-AMD3465 [68], ^68^Ga-labeled highly specific targeted contrast agent ^68^Ga-CPCR4-2 [69], ^68^Ga-DOTA-4-FBn-TN14003 [70], ^18^F-FP-labeled Ac-TC14012 [71], ^68^Ga-CCIC16, N-^11^C-Methyl-AMD3465 [72], ^68^Ga-pentixafor [73–75], ^68^Ga-NOTA-NFB [76], ^18^F-NOTA-T140 [77]. Among these miscellaneous tracers, the most noted tracer backbone was pentixafor, a synthetic peptide analogous to CXCL12 that specifically binds to CXCR4. Preclinical studies showed that pentixafor-based PET-tracer ^68^Ga-Pentixafor exhibited high tumor-to-background contrast and outstanding CXCR4 specificity. To date, ^68^Ga-Pentixafor has been evaluated in multiple types of hematologic and solid malignancies and demonstrated promising diagnostic and treatment assessment values in multiple myomas, small-cell lung cancer, and several other types of hematologic malignancies (Fig. 3). ^68^Ga-Pentixafor presented superior background contrast and lesion detection sensitivity than ^18^F-FDG in several types of hematological cancers. ^68^Ga-Pentixafor imaging has the potential for disease follow-up, treatment response prediction, and patient selection for the directed therapy [73–75, 78–99] (Table 1).

**Table 1:** ^68^Ga-Pentixafor imaging in hematological and solid cancer patients

Tumor type	Authors	Year	Study Type	Sample size	Main findings
Lymphoproliferative cancers	Wester et al. [73]	2015	Proof-of-concept	4	Validation of the feasibility of ^68^Ga-Pentixafor in cancer patients Excellent imaging in TCL with NSCLC, DLBCL, CLL ,MM
MM	Herrmann et al. [74]	2015	Pilot-study	5	Biodistribution and dosimetry of ^68^Ga-Pentixafor
	Philipp et al. [75]	2015	Pilot-study	14	Complementary to ^18^F-FDG in lesion detection
	Lapa et al. [78]	2017	Retrospective	35	^68^Ga-Pentixafor positivity was a negative prognostic factor Complementary to ^18^F-FDG in lesion detection
	Pan et al. [79]	2020	Prospective	30	^68^Ga-Pentixafor was promising in imaging diagnosis of MM
	Shekhawat et al. [80]	2022	Retrospective	34	^68^Ga-Pentixafor was apt to MM diagnosis and staging
MCL	Mayerhoefer et al. [81]	2021	Prospective	22	^68^Ga-Pentixafor revealed higher detection rates and better tumor-to-background contrast than ^18^F-FDG
	Mayerhoefer et al. [82]	2023	Retrospective	16	^68^Ga-Pentixafor presented superior treatment assessment than MRI
MZL	Duell et al. [83]	2021	Retrospective	16	^68^Ga-Pentixafor is promising in imaging diagnosis of MZL
CNSL	Herhaus et al. [84]	2020	Proof-of-concept	11	^68^Ga-Pentixafor is promising in imaging diagnosis of CNSL ^68^Ga-Pentixafor has treatment response prediction value
	Starzer et al. [85]	2021	Prospective	7	^68^Ga-Pentixafor is promising in monitor of CNSL
	Chen et al. [86]	2022	Retrospective	26	^68^Ga-Pentixafor is superior in detection of CNSL lesions than ^18^F-FDG
WM/LPL	Luo et al. [87]	2019	Prospective	17	^68^Ga-Pentixafor is a promising agent for WM/LPL detection
	Pan et al. [88]	2021	Prospective	15	^68^Ga-Pentixafor is superior in post chemotherapy response assessment than ^18^F-FDG
MALT	Haug et al. [89]	2019	Prospective	36	^68^Ga-Pentixafor is promising in MALT detection
	Mayerhoefer et al. [90]	2022	Prospective	46	^68^Ga-Pentixafor is promising in treatment assessment of MALT
Glioblastoma	Lapa et al. [91]	2016	Pilot-study	15	^68^Ga-Pentixafor has potential for patient selection of CXCR4 directed treatment
	Jacobs et al. [92]	2022	Pilot-study	7	^68^Ga-Pentixafor imaging is not strictly correlated to IHC staining
Solid and hematologic	Buck et al. [93]	2022	Retrospective	690	High image contrast in hematologic, SCLC, adrenocortical cancer
Solid cancers	Vag et al. [94]	2016	Proof-of-concept	21	Aid tumor diagnosis
	Werner et al. [95]	2019	Retrospective	19	Aid tumor diagnosis
	Serfling et al. [96]	2022	Retrospective	90	No tumor sink effect observed in solid cancer patients
	Hartrampf et al. [97]	2023	Retrospective	50	Interobserver agreement rates of ^68^Ga-Pentixafor imaging
Breast cancer	Vag et al. [98]	2018	Retrospective	18	Not suitable for breast cancer general diagnosis
NEC	Weich et al. [99]	2021	Retrospective	11	Aid tumor diagnosis

TCL, T-cell lymphoma; NSCLC, non-small cell lung cancer; DLBCL, diffuse large B-cell lymphoma; CLL, chronic lymphocytic leukemia; MM, multiple myeloma; MCL, Mantle cell lymphoma; MZL, marginal-zone lymphoma; CNSL, central nervous system lymphoma; WM/LPL, waldenström macroglobulinemia/lymphoplasmacytic lymphoma; MALT, gastric mucosa-associated lymphoid tissue lymphoma; NEC, neuroendocrine carcinomas

#### EpCAM

EpCAM was a classic epithelial cell marker ubiquitously expressed in epithelial cells and stem cells. It was once proposed as a CSC marker due to its tumor-promoting function and involvement in stemness regulating pathways, including Wnt/β-catenin and TGF-β/SMAD pathways [100]. Catumaxomab, a trifunctional bispecific antibody targeting EpCAM-expressing tumor cells, CD3-expressing T cells, and antigen-presenting cells, was approved for treating malignant ascites of patients with positive EpCAM. An initial attempt for PET imaging of EpCAM expressing tumors was achieved in A-431 tumor-bearing athymic mice using ^68^Ga-labelled HBED-CC scFv42_9_, an HBED chelated diabody [101]. Recent researchers tested PET imaging of EpCAM in MDA-MB-231 tumors with a ^64^Cu−aptamer radiotracer [102]. Sergey et al. adopted a strategy in which a designed ankyrin repeat protein Ec1 was employed for specific EpCAM-binding. They evaluated the combination of two different positional labeling of Ec1 and four different radioactive nuclides ^68^Ga, ^111^In, ^57^Co, and ^125^I. According to their results, ^125^I-PIB-H6-Ec1 outcompeted all other tracers. It demonstrated the most outstanding tumor-to-background contrast as early as 3 to 6 h post-infection in tumor models of pancreatic cancer, ovarian cancer, and triple-negative breast cancer [103–105].

### Nanobiotechnology-optimized CSC eradication

Due to the innate characteristics of CSCs, conventional therapies such as chemotherapy and radiotherapy that effectively targeted bulk tumor cells often failed to eradicate CSCs, which led to CSC enrichment. Moreover, CSC phenotypes were somewhat diversified by chemotherapeutic or radiotherapeutic modulation of both CSC and CSC niches. The post-conventional therapeutic enrichment and diversification of CSCs would lead to inevitable tumor relapse and metastasis. Therefore, eradicating CSCs might require combining strategies, including conventional therapy sensitization, CSC differentiation modulation, CSC niche targeting, and tumor immunity activation. Nanobiotechnology has several advantages in treating CSCs, including precise targeting, high-dose drug delivery, multiple drug delivery, controlled drug release, and artificial antigenicity [106]. Here, we reviewed recent advances in nanobiotechnology-optimized CSC-targeted treatment with in vivo validation in routine clinical cancer management including cytotoxic therapy, photodynamic therapy, immunotherapy, and radioimmunotherapy. Strategies regarding sonodynamic therapy and ablation therapy were formerly explicitly reviewed [107, 108].

#### Cytotoxic therapy

One of the major obstacles to CSC treatment was the innate resistance of CSC to conventional cytotoxic therapy. Several strategies were put forward: increase the drug dosage of specific CSC; modulation of resistance-associated genes or pathways; induction of CSC differentiation followed by conventional cytotoxic therapy; boosting various types of cellular death such as ferroptosis. However, specific CSC modulation requires precise delivery of modulators. To this end, many researchers studied the feasibility and anti-CSC efficacy of using CSC-targeted nanomaterial-based drug delivery systems. For CSC-specific targeted cytotoxic agents delivery, Ning et al. designed CD133 antibody conjugated nanoparticles to deliver SN-38, the active form of water-soluble camptothecin-11 (CD133Ab-NPs-SN-38) for CD133^+^ CSC targeting tested in HCT116 human colorectal cancer xenografts [109]. Li et al. synthesized HA-grafted all-trans-retinoic acid cationic nanoparticle (HA-eNPs/ATRA) and validated its anti-CSC/cancer effect in CD44 enriched B16F10 murine melanoma tumor model [110]. Wang et al. tested their doxorubicin-loaded HA-Lys-La nanoparticles (X-NP-DOX) in human breast cancer cell line MCF-7 bearing mice. They found significantly increased drug enrichment in the tumor. X-NP-DOX significantly reduced the tumorgenicity of CSC and exhibited an enhanced anti-tumor effect [111]. For CSC differentiation treatment, Geng et al. utilized a hypothesis-free method screening out nanoparticle C6NP, which has the most remarkable ability to induce CSC differentiation and sensitize CSC to conventional cytotoxic therapy [112]. A recent study by Wu et al. reported amplified ferroptosis through an iron oxyhydroxide-based nanosystem (FeOOH/siPROM2@HA) using a “three-pronged” strategy [113].

#### Photodynamic therapy

Photodynamic therapy exploits the reactive oxygen species generated by light-activated photosensitizers to eliminate cancer cells. Since FDA approved clinical photodynamic therapy in dermatological tumors, studies into photodynamic therapy aiming at effect enhancement and scope expansion have bloomed. Though compared to conventional therapy, precise targeting and controlled activation significantly reduced the systematic side-effects of photodynamic therapy, it faced limitations such as low tumor specificity and hypoxic tumor microenvironment. A combination of nano-drug delivery systems and photodynamic therapy might increase the overall systematic solubility, stability, target specificity, and biocompatibility and achieve synergetic effects through the co-delivery of photosensitizers and cytotoxic agents. The fundamental element of CSC targeting photodynamic therapy strategy usually includes a CSC targeting nanomaterial or CSC ligand, a photosensitizer, and sometimes added chemotherapeutic or CSC modulating agents. However, the cytotoxic effects of primordial photodynamic strategies against CSCs were far from satisfactory due to the original hypoxic state of CSCs and their high tolerance to reactive oxidative damage. A few studies focused on CSC targeting through nano-drug carrier-mediated photodynamic therapy and achieved particular progress.

One of the advantages of photodynamic therapy was that loaded drugs could be precisely released upon photo-activation. Ren et al. reported that by near-infrared activation of the sequential release of co-delivered miR-21 inhibitor and doxorubicin, breast cancer cells and CSCs were synergistically inhibited both in vitro and in vivo. At the same time, the synergetic effect of which was failed by simple co-delivery of the two drugs [114]. Exploiting photodynamic activation, Lee et al. constructed a CD44 targeting ROS-cleavable thioketal-SN38 conjugated hyaluronan-cholesterol nanoparticles. They successfully validated its drug delivery, drug release, and therapeutic effects using taxol-resistant ovarian cancer cell line HEY-30 bearing BALB/c [115]. Jung et al. developed a multi-functional CSC targeting system CA9-BPS-Cu(II), which combined chemo- and photodynamic effects with an acetazolamide-based approach [116]. In vitro, spheroid formation assay validated the cytotoxic effects of CA9-BPS-Cu(II) to CD133^+^ and CD44^+^/CD24^−^ MDA-MB-231 breast cancer cells. The CSC marker ALDH and stemness-associated transcription factors such as OCT4, Nanog, SOX9, and Stat3 were significantly inhibited.

To cope with the hypoxic environment around CSC, Ning et al. designed a CD44 targeting type one aggregation-induced emission photosensitizer-loaded biomimetic mesoporous organosilicon nano-system to prevent cancer recurrence after radiotherapy [117]. Their nano-system achieved in vivo CSC targeting and significant tumor inhibition combined with radiotherapy. The proportion of in vivo CSC markers, including ALDH and CD133, significantly decreased. Considering the possible reduction of photo-responsiveness when conjugating photosensitizer directly to a specific antibody, Wang et al. proposed non-specific CSC targeting through a ribosome targeting [118]. In case CSCs were found to resist photodynamic therapy, novel strategies were investigated. Photochemical internalization, a highly efficient method of macromolecule delivery, especially for toxins that tended to be trapped in endosomes, had the potential to circumvent CSC photodynamic therapy resistance. Bostad et al. constructed a CD133 targeting photochemical internalization system PCI_AC133−saponin_ and validated its CSC cytotoxicity in vitro and in vivo [114]. Multifunctional nanoplatforms with PET imaging ability and anti-cancer effect were extensively studied to fill the gap between simultaneous imaging and NIR treatment. Dong et al. designed a pH, HAse, and NIR responsive nano platform, MoS2-PEI-HA, which could be conjugated with either doxorubicin or ^64^Cu-NOTA. With ^64^Cu-NOTA labeled MoS2-PEI-HA, tumor uptake and dosages could be more specifically evaluated pre-treatment, and with the NIR responsiveness of DOX@ MoS2-PEI-HA, the release rate of DOX could reach up to 77.4% [119].

#### Immunotherapy

**Fig. 4:**
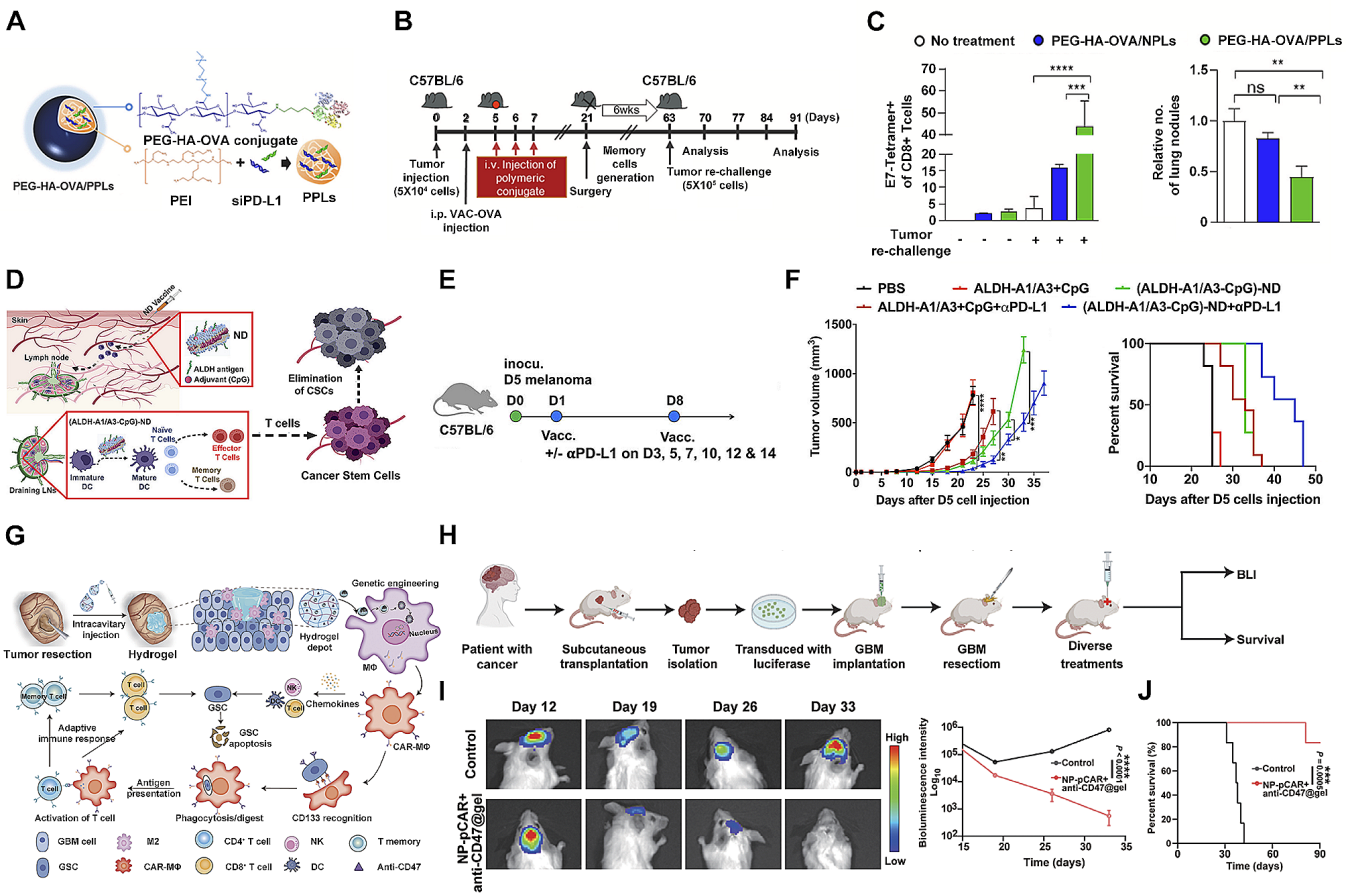
Schematic strategies and in vivo validation of nano-biotechnological optimized cancer stem cell-targeted immunotherapy. A Structural illustration of PEG-HA-OVA/PPLs. B and C Long-term in vivo validation of lung cancer protection by PEG-HA-OVA/PPLs [124]. D Graphical mechanism of vaccination against ALDH^+^ cancer stem cell. E and F Therapeutic effect of ALDH-ND against melanoma D5 tumor model [125]. G Schematic illustration of hydrogel application, CAR-MΦ conversion, cancer stem cell antigen presentation, adaptive immune response activation, and natural killing. H, I and J In vivo simulation of the post-surgical treatment effect of nano porter hydrogel [127]. HA, hyaluronic acid; OVA, ovalbumin; ND, nanodisc. All panels were reproduced with permission

Immunotherapy mobilizes immune cells to attack cancer cells. Standard immunotherapy includes immune checkpoint inhibitors, immune system modulators, virus therapy, cancer vaccines, and engineered immune cell transfer. Despite high cancer targeting specificity and long-lasting effects in responsive patients, there were still challenges for immunotherapy to target CSC, which include heterogeneity, immune evasion, and microenvironment remodeling. The advantages of nanotechnology, including multiple drug delivery, multi-specific targeting, controlled drug release, and environmental responsiveness, can augment immunotherapy when dealing with these challenges for targeting CSC. Here, we systematically reviewed some up-to-date research on nanotechnology-assisted immunotherapeutic approaches targeting CSCs, including immune checkpoint inhibitors, cancer vaccines, and engineered immune cells (Fig. 4).

Immune checkpoint inhibitors for cancer treatment have been one of the most active research areas of cancer immunotherapy. Advances in nanobiotechnology largely boosted the safety and efficacy of immune checkpoint inhibitors [120, 121]. While immune checkpoint inhibitors could eliminate the bulk tumor, CSCs demonstrated resistance to immune checkpoint inhibitors via expressing extremely high levels of immune checkpoint proteins, including PD-L1, CTLA-4, LAG-3, and TIM-3. However, administration of free immune checkpoint inhibitors would cause systematic adverse effects. Therefore, CSC-specific delivery of adequate immune checkpoint inhibitors would increase potency and reduce side effects. Several groups had focused on PD-L1 inhibitor delivery to CD44^+^ breast cancer cells. To supplement the anti-CSC effect in conventional chemo-/immunotherapy, Lang et al. developed a double-layered nanodevice loaded with PD-1/PD-L1 inhibitor HY19991 and anti-CSC agent thioridazine on the surface and paclitaxel inside. The nano delivery system delivers the loaded drugs to the tumor by passive targeting, and sequential release of the cargoes was triggered in response to the tumor microenvironment and endocytosis of the tumor cells [122]. The strategy of more recent studies to enhance PD-1/PD-L1 inhibitor potency on CD44^+^ CSC mostly involved (a) PD-1/PD-L1 inhibitor, (b) active targeting either through CD44 ligands including hyaluronic acid (HA) and chondroitin sulfate (CS), or anti-CD44 aptamer; (c) immunogenic antigen enhancement either by combination with chemotherapy or targeted therapy or implement of immunogenic foreign antigens. Kim et al. used HA as a CD44 targeting ligand to deliver PD-L1 siRNA for PD-L1 inhibition (PEG-HA-PPL) and ovalbumin (OVA) for immunogenic enhancement (PEG-HA-OVA) [123]. Cheng et al. equipped their paclitaxel/chloroquine delivery nanoparticle with CD44 targeting agent CS, immunogenic OVA, immunopotentiator CpG, and PD-L1 inhibitor atezolizumab (CpG + OVA + PTX + CQ-N/A). According to their results, PEG-HA-PPL/OVA, CpG + OVA + PTX + CQ-N/A, and all elicited tumor-reactive T cell-dependent anti-tumor effect and long-term protection in murine models [124].

However, to target CSC characterized by intracellular CSC markers such as ALDH, SOX2, and Nanog, direct biomarker-targeting through mAb was not feasible. In this case, immunogenic epitopes of corresponding CSC markers could be utilized to develop a specific therapeutic cancer vaccine. Alireza et al. developed a nanodisc (ND) vaccine delivery system to deliver antigenic ALDH1-A1 and ALDH1-A3 epitopes to antigen-presenting cells to induce specific ALDH^+^ CSC T cell response. They successfully constructed (ALDH-A1/A3-CpG)-ND of 9 to 13 nm, confirmed the lymphatic delivery and uptake of antigen in dendritic cells, B cells, and macrophages, validated T cell activation in vivo, and proved the robust therapeutic effects of (ALDH-A1/A3-CpG)-ND vaccine combined with anti-PD therapy in murine breast cancer models [125]. Their subsequent research identified four more SOX2 and Nanog epitopes with potential for vaccine development [126].

Adaptive chimeric antigen receptor (CAR) immune cell transfer is a more specific and potent way to target CSC. Artificially engineered CAR-expressing immune cells, including CAR-T, CAR-NK, CAR-DC, and CAR-M, could directly bind to cancer cells, significantly increasing cytotoxicity or antigen-presentation. Recent work by Chen et al. provided a simple way to induce local CD133-specific CAR-M through nanopore-hydrogel in post-surgical areas. They synthesized a CD68 promoter-driven anti-CD133 nanopore CAR (pCAR) and loaded to hydrogel coated with brain ECM-derived laminin/immune-stimulating peptides and citraconic anhydride–modified dextran to form macrophage-targeted NP-pCAR. NP-pCAR successfully induced anti-CD133 CAR-expressing macrophages, promoted phagocytosis of CD133^+^ cancer cells by CAR-M, activated T cell cytotoxicity, and elicited T cell memory [127].

#### Radioimmunotherapy

Radiopharmaceutical therapy is a type of cancer treatment that provides cancer-targeted radiation through the bloodstream via remedial radiopharmaceuticals. Radiopharmaceuticals consist of a radionuclide that emits radiation and a targeting ligand. Although radiopharmaceuticals were not as extensively used as other therapies such as surgery and chemotherapy, the number of radiopharmaceuticals designated for cancer diagnosis and treatment exceeds one-third of the current FDA-approved radiopharmaceuticals. In addition to the well-established application of iodine ^131^I-sodium iodide in carcinoma of thyroid and ^223^radium-dichloride in prostate cancer, more and more novel radiopharmaceuticals both for diagnosis and for treatment are getting approved by the FDA or undergoing clinical trials. Compared to conventional radiotherapy, radiopharmaceuticals have higher penetration capacity, can target primary and metastatic lesions in one shot, minimize average tissue exposure to radiation, and can even activate an anti-tumoral immune response when properly edited. Here, we mainly review how radiopharmaceuticals can be tailored to target CSCs.

CSCs represent only a tiny fraction of cancer cells and are resistant to chemotherapy, radiotherapy, and immunotherapy. Targeting CSCs through CSC-specific epitope-directed radionuclides is a promising therapeutic strategy. Several groups have tried to achieve high precision of CSC targeting based on current known CSC markers. In the past few years, ^131^I-antiAC133.1mAb, ^131^I-antiCD44mAb, ^131^I-antiCD44V6mAb, and ^177^Lu-antiCD44V6mAb were reported to have tumor inhibition effects in colorectal cancer HCT116 bearing xenografts [128–130]. However, since CD44 and CD133 were not solely expressed in CSC but also in many normal stem cells, the clinical validation of CD44 and CD133-directed therapy might meet foreseeable challenges. Until now, none of the CD44-directed radiopharmaceuticals has made it to clinical trial since the termination of two clinical trials of CD44v6 targeting antibody-drug conjugate bivatuzumab mertansine due to severe epidermal necrolysis.

Among the currently known CSC markers, CXCR4-directed radiopharmaceuticals achieved ground-breaking successes. In-human evaluation of CXCR4-directed radioligand therapy was performed in small-scale patients with multiple myoma [131, 132], advanced diffuse large B-cell lymphoma as part of the conditioning regimen before allogeneic stem cell transplantation [133], acute myeloid leukemia [134], advanced T-cell lymphoma [135]. The evaluated treatment strategy mainly involved selecting patients via ^68^Ga-Pentixafor imaging, pre-therapeutic dosimetry assessment through ^177^Lu-pentixather, and precision radioactive therapy by ^90^Y-pentixather. Almost all studies evaluating in-human ^68^Ga-Pentixafor imaging performance reported outstanding tolerance and zero major adverse events. However, regarding radiopharmaceutical therapy, non-negligible side effects were observed. A retrospective study assessed the side effects of radionuclide conjugated-pentixather in twenty-two patients with heavily pretreated hematopoietic cancers. The mild and slight probability of adverse events relating to drug elimination sites, including kidney and liver, added to the feasibility of pentixather-directed radiopharmaceutical therapy. However, hematopoietic side effects, the most frequent and serious ones, would limit pentixather-directed radiopharmaceutical therapy to hematopoietic stem cell transplantation setting [136]. With promising clinical translation pentixafor and pentixather, a novel generation of pentixafor and pentixather with higher specificity was studied. Replacement of the linker AMBS, which puts DOTA and CXCR4 binding core CPCR4 in pentixafor and iodoCPCR4 in pentixather together, by r-α-ABA would increase the hCXCR4 affinity of novel compounds tenfold. Although the novel ^68^GaDOTA-r-a-ABA-CPCR4 and -iodoCPCR4 did not show superior imaging performance than ^68^Ga-Pentixafor, the 48 h tumor-to-background ratios of ^177^Lu-DOTA-r-a-ABA-CPCR4 doubled even quadrupled compared to that of ^177^Lu-Pentixather [137]. The theranostic value of ^177^Lu-DOTA-r-a-ABA-CPCR4 as a second generation of CXCR4-directed radiopharmaceutical therapy was worth further evaluation.

## Conclusion

CSCs represent both fundamental driving forces and therapeutic targets of tumoral carcinogenesis, tumor evolution, progression, and recurrence. Leveraging the characteristics of CSCs for early cancer diagnosis, dynamic tumor surveillance, and enhancement of therapeutic effects are facilitated by updating nanobiotechnologies. Though the field has accumulated fruitful results with clinical translational potential in the foreseeable future, tackling CSC remains challenging. The rarity, heterogeneity, and plasticity of CSCs call for ultrasensitive detection methods and multi-target targeting strategies. What we know about CSCs is still limited in varying genetic and epigenetic contexts of different cancer types. With an evolving understanding of the underlying mechanisms of maintenance of stem-like properties, the CSC niche, CSC-cancer evolution, the cross-talks between CSCs and tumor-microenvironment, novel markers, cellular targets, and cellular interaction modes can be exploited for holistic CSC targeting.

## Data Availability

No datasets were generated or analysed during the current study.

## Abbreviations

CSC: Cancer stem cell
CAR: chimeric antigen receptor
CS: chondroitin sulfate
CTC: circulating tumor cell
ctDNA: circulating tumor DNA
FDA: Food and Drug Administration
FET: field-effect transistor
FRET: Förster resonance energy transfer
GBM: glioblastoma
HA: hyaluronic acid
MRI: magnetic resonance imaging
ND: nanodisc
NSCLC: non-small cell lung cancer
OVA: ovalbumin
PET: positron emission computed tomography
SCLC: small cell lung cancer
SERS: surface enhanced Raman scattering
SPR: surface plasmon resonance
USPIO: ultrasmall superparamagnetic iron oxide
^18^F-FDG: ^18^F-Fluoro-2-deoxy-D-glucose
^18^F-FSPG: (S)-4- (3- 18 F-Fluoropropyl)-L-Glutamic Acid
